# Malignant skin adnexal tumor of the finger with late metastases

**DOI:** 10.1016/j.radcr.2025.11.083

**Published:** 2026-01-06

**Authors:** Mohammad Hussain Erfani, Surenth Nalliah, Idris Abdulrahman Abdullah Akreyi, Morten Smærup Olsen

**Affiliations:** a Department of Radiology, Aalborg University Hospital, Aalborg, Denmark; b Department of Clinical Medicine, Aalborg University, Aalborg, Denmark

**Keywords:** Malignant skin adnexal tumor, MCATs, Spiradenocylindroma, Brain metastasis, PET/CT, MRI

## Abstract

Malignant cutaneous adnexal tumors (MCATs) are rare neoplasms with unpredictable metastatic behavior and limited radiologic characterization. We report a case of malignant spiradenocylindroma initially evaluated with ultrasound, which demonstrated a pear-shaped heterogeneous subcutaneous structure with slight peripheral hyperemia and a small fluid-filled component in the distal phalanx of the left third finger, interpreted as a possible post-traumatic hematoma or foreign-body reaction consistent with the clinical history. Three years later, magnetic resonance imaging (MRI) revealed an infiltrative, contrast-enhancing mass with adjacent bone destruction, confirming a malignant process. After amputation, the patient remained disease-free for 8 years before developing neurological symptoms. Brain MRI showed an unusual multilobulated cystic metastasis, and positron emission tomography combined with computed tomography (PET/CT) demonstrated metabolically active pulmonary metastases. The patient ultimately experienced a fatal outcome. This case highlights the diagnostic challenges in early presentation and underscores the potential for delayed distant metastases in MCATs while expanding the radiologic spectrum of intracranial involvement to include rare multilobulated cystic presentations.

## Introduction

Malignant cutaneous adnexal tumors (MCATs) are rare and heterogeneous neoplasms arising from eccrine, apocrine, sebaceous, or follicular structures [[1], [2], [3]]. Although most adnexal tumors are benign, a subset may progress from indolent growth to aggressive behavior with distant metastases [2,4], most commonly involving the lungs, liver, or bones; brain involvement is exceptionally rare [5,6]. Malignant spiradenocylindroma is an uncommon subtype that can arise through malignant transformation of a spiradenoma or cylindroma [7]. Diagnosis is often delayed because of overlapping histological and radiological features that complicate early recognition [8,9]. This case contributes to the scarce literature on MCATs with late metastatic spread and reinforces the need for continued clinical and radiological awareness when evaluating rare skin tumors.

## Case presentation

**Fig. 1:**
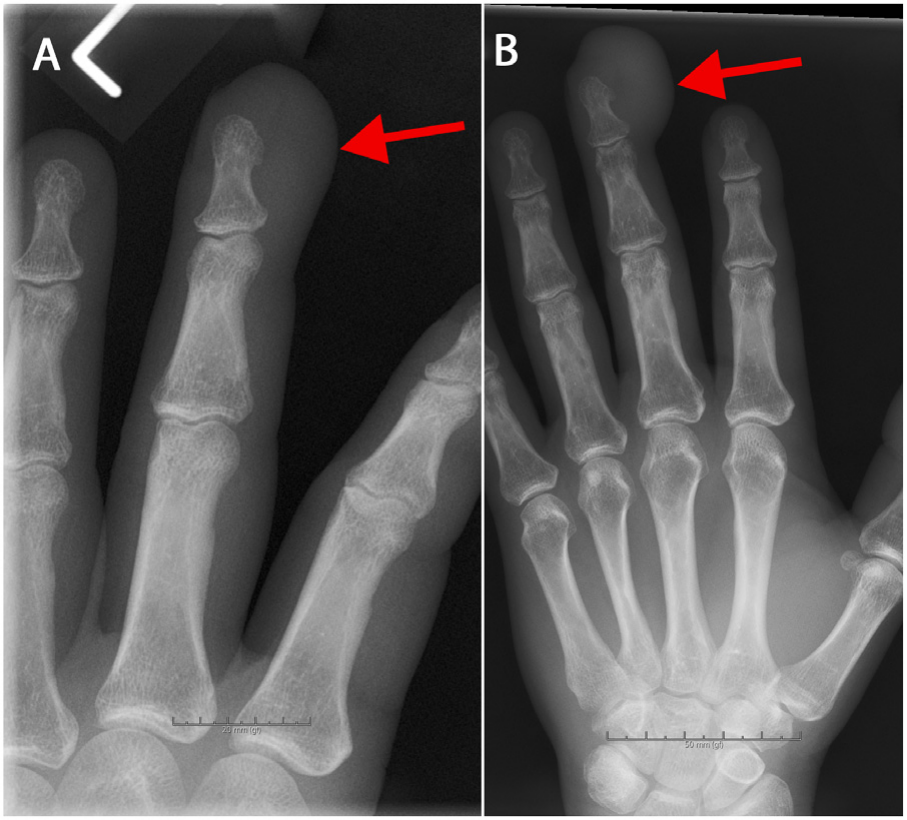
(A) Plain radiograph of the left third finger (2012) shows soft tissue swelling at the fingertip, without evidence of bone erosion or a visible foreign body (red arrow). (B) Follow-up radiograph of the same finger in 2015 demonstrates progression of soft tissue swelling compared with the 2012 image, along with subtle bone involvement, still without detectable foreign body (red arrow).

**Fig. 2:**
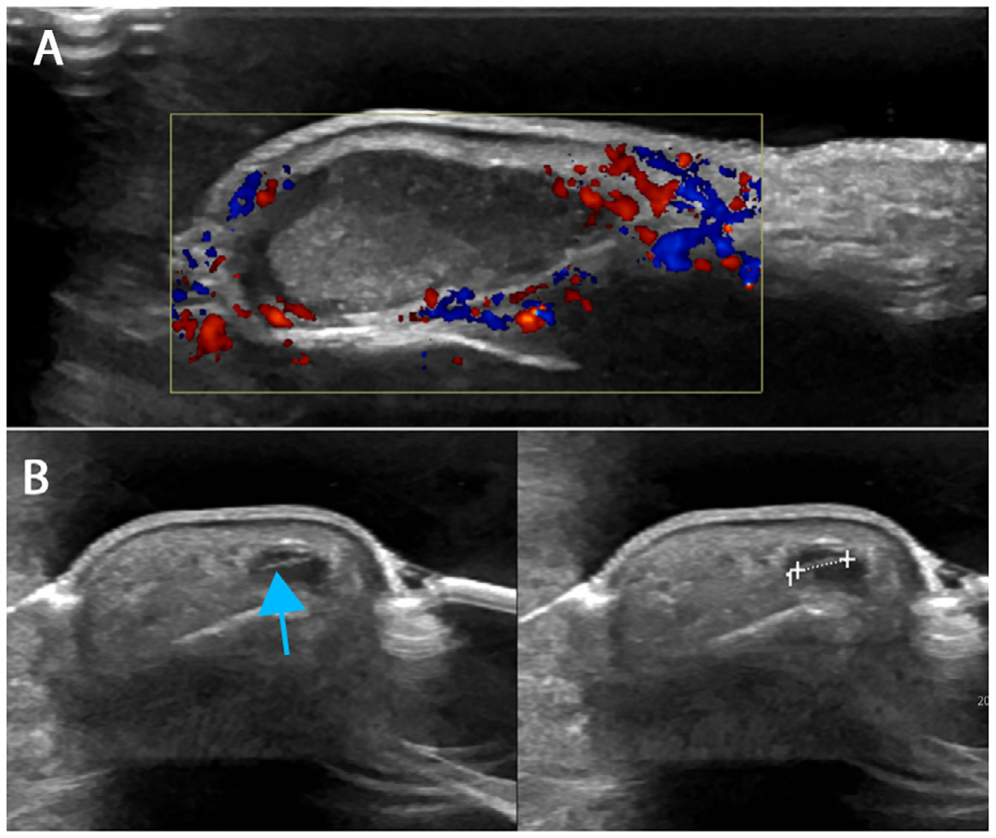
(A) Longitudinal and (B) transverse ultrasound images of the left third finger (2012) demonstrate a hypoechoic, pear-shaped subcutaneous lesion with mixed echogenicity and increased perifocal doppler activity within the surrounding soft tissues. A linear hyperechoic focus, suggestive of a retained foreign body, is identified at a depth of 5-6 mm (blue arrow).

A 63-year-old man, previously healthy and employed in technical service management, presented in early 2012 with localized swelling in the distal phalanx of his left third finger after a presumed gardening injury. Plain radiograph of the finger revealed soft tissue swelling without bone erosion or any radio-opaque foreign body visible (Fig. 1A). Ultrasound of the left third finger was performed by a general radiologist upon clinical referral due to a presumed gardening-related foreign body injury. The examination was interpreted as demonstrating a pear-shaped, heterogeneous subcutaneous structure located radially to the distal phalanx pulp region, which showed slight peripheral hyperemia, and a small fluid-filled area containing a suspected 2-3 mm cylindrical foreign body. The findings were interpreted as consistent with a possible post-traumatic hematoma or foreign body reaction (Fig. 2). Surgical exploration of the finger resulted in drainage of purulent fluid, and it was considered possible—though unconfirmed—that a foreign body might have been expelled with the discharge. No tissue or fluid samples were sent for pathological examination.

**Fig. 3:**
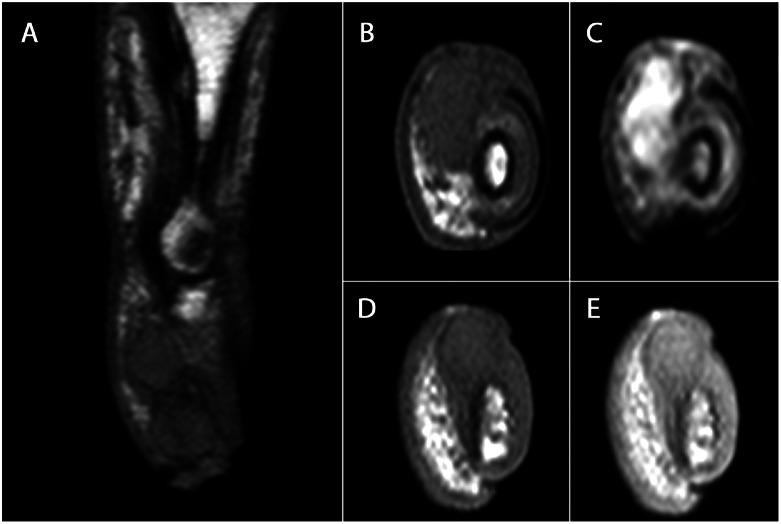
(A) Sagittal T1-weighted MRI of the left third finger (2015) demonstrates 2 adjacent, well-defined soft-tissue nodules in the distal phalanx, each approximately 6 mm in diameter and distinguishable from one another. (B-C) Axial T1-weighted (B) and fluid-sensitive (C) sequences demonstrate the proximal nodule. (D-E) Axial T1-weighted (D) and post-contrast T1-weighted (E) images show the distal nodule with moderate enhancement and evidence of invasion into the underlying phalangeal bone and overlying epidermis.

In 2015, the patient returned with a painless, slowly enlarging mass at the same site. A new plain radiograph of the left third finger demonstrated progressive soft tissue swelling compared with previous imaging, as well as subtle bone involvement (Fig. 1B). Magnetic resonance imaging (MRI) of the affected finger revealed 2 adjacent nodular masses in the distal phalanx with low-to-intermediate T1 signal intensity, hyperintensity on fluid-sensitive sequences, moderate enhancement, and evidence of bone invasion (Fig. 3). Malignancy was verified after biopsy, and therefore partial excision was performed, followed by amputation at the proximal interphalangeal joint due to positive margins. Histopathology revealed a malignant adnexal tumor with mixed features of spiradenoma and cylindroma.

**Fig. 4:**
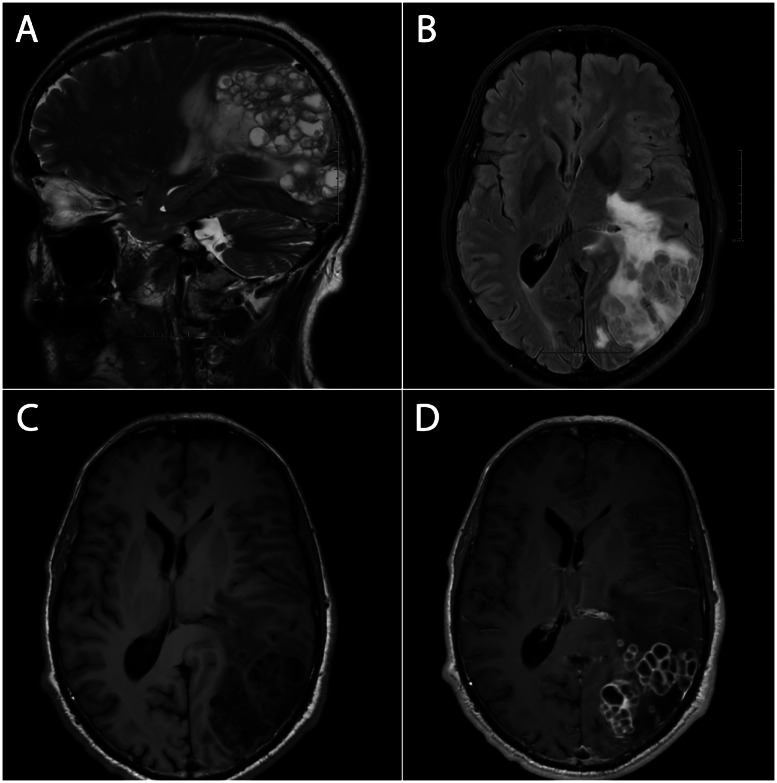
(A) Sagittal T2-weighted MRI demonstrates the multilobulated architecture and the extent of the cystic lesion in the left parieto-occipital region. (B) Axial T2-weighted FLAIR sequence demonstrating mass effect and surrounding vasogenic edema. (C-D) Axial T1-weighted pre-contrast (C) and T1-weighted post-contrast (D) MRI depicts a multilobulated, bubbly-appearing cystic lesion with rim enhancement and associated mass effect.

**Fig. 5:**
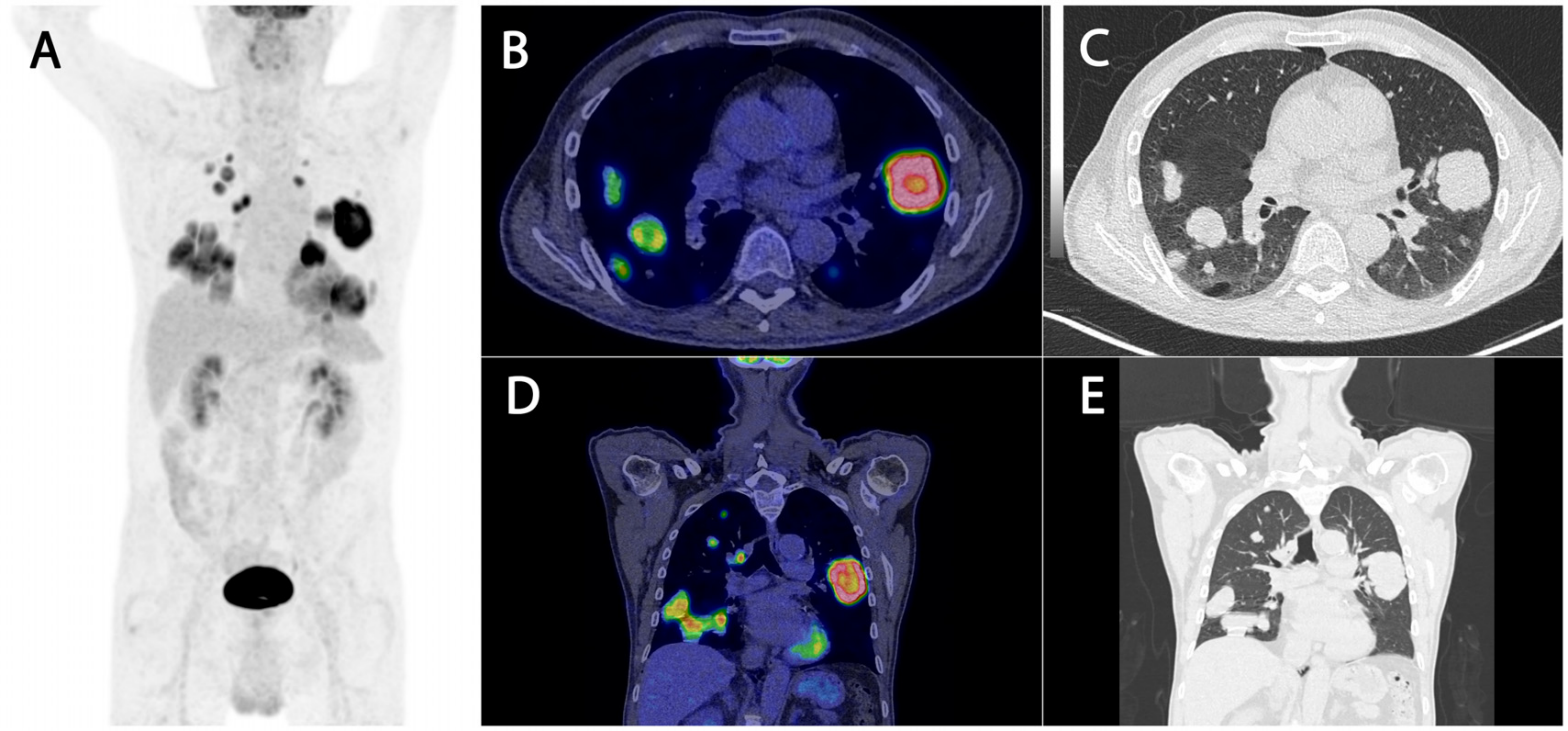
(A) Whole-body maximum intensity projection (MIP) PET image reveals multiple large pulmonary nodules bilaterally and mediastinal lymphadenopathy. The largest nodule, located in the right lower lobe, measures 7 cm. (B-E) Axial (B-C) and coronal (D-E) CT and corresponding positron emission tomography/computed tomography PET/CT images from 2023 demonstrate intense fluorodeoxyglucose (FDG) uptake in multiple pulmonary nodules and right hilar lymph nodes, consistent with metabolically active metastatic disease.

The patient remained asymptomatic until July 2023, where he developed cognitive impairment, balance issues, and speech disturbances. The possibility of a stroke was suspected, and brain MRI demonstrated a multilobulated cystic lesion involving the left parietal and occipital lobes, with rim enhancement and surrounding edema (Fig. 4). Computed tomography (CT) of the thorax revealed bilateral, confluent pulmonary nodules up to 7 cm in diameter and mediastinal lymphadenopathy. Positron emission tomography combined with computed tomography (PET/CT) showed intense fluorodeoxyglucose (FDG) uptake in the lung lesions and right hilar nodes (Fig. 5). A CT-guided biopsy of the right lung mass confirmed metastasis from the original adnexal tumor.

He was treated with whole-brain radiotherapy (30 Gy in 10 fractions) and corticosteroids (prednisolone 50 mg/day). His condition deteriorated, and he died in January 2024 under palliative care.

## Discussion

Radiological imaging for MCATs is indispensable in initial evaluation, staging, and monitoring of progression, particularly in atypical or delayed metastatic patterns [5,9].

The initial ultrasound in 2012 was interpreted as a possible post-traumatic hematoma or foreign body reaction, consistent with the clinical history at the time. Sonographically, the lesion appears as a nonspecific, pear-shaped, predominantly solid subcutaneous pathologic lesion with slight peripheral hyperemia. In retrospect, such a presentation might have warranted short-term follow-up to ensure resolution. Previous studies have shown that clinical context can subtly influence radiologic interpretation, and that anchoring and framing based on clinical expectations may narrow diagnostic reasoning and limit consideration of alternative differentials [10,11]. In this case, the initial assessment was appropriately aligned with the information provided, but the suspicion of a foreign body likely functioned as a cognitive anchor.

The MRI findings in adnexal tumors are rarely reported and mostly appear as isolated case reports, meaning that imaging characteristics across subtypes remain poorly defined. MRI is well suited for assessing soft-tissue lesions and signs of aggressive behavior, helping guide diagnostic work-up towards biopsy and histopathology as the gold standard, and pre-operative planning [9,12]. In the present case, the MRI features—destruction of the distal phalanx, cutaneous involvement, lack of a capsule and marked contrast enhancement—strongly indicate a malignant soft-tissue lesion.

Only a few studies have described latency periods in metastatic MCATs, with most cases developing metastases within a few years of initial treatment [5,6]. The occurrence of distant metastasis 8 years after surgery in our patient highlight the importance of long-term follow-up.

The use of PET/CT is valuable in these rare skin malignancies, aiding in the detection of occult disease, guiding biopsy, and monitoring treatment response [9,13]. In our case, PET/CT demonstrated intense FDG-activity in pulmonary nodules and right hilar lymphadenopathy without evidence of further systemic spread, and histopathology confirmed these lesions as MCAT metastases. The brain lesion was not biopsied, but the presence of pulmonary metastases strongly suggests metastatic spread from the known MCAT to the brain. Despite the few reported cases of MCAT brain metastases that involved solid lesions, partially cystic features, and fully cystic configurations [5,6,14,15], the multilobulated cystic configuration observed in our case highlights the potential spectrum of radiologic appearances in MCAT metastases.

Treatment options for metastatic MCATs are limited. Prognosis is generally favorable for localized disease but becomes poor with nodal or distant metastases [4,13]. Surgical excision remains the standard treatment for localized tumors [4,7], whereas systemic and targeted therapies show inconsistent results [5,13], and immunotherapy remains experimental [16].

## Conclusion

This case highlights the risk of delayed distant metastases from MCAT, with a potentially fatal outcome. Early clinical suspicion combined with diagnostic imaging and timely histopathologic evaluation, together with long-term clinical follow-up, is essential for accurate diagnosis and optimal management in high-risk MCATs.

## Patient consent

As the patient was deceased, consent for publication of this case report, including any accompanying images and clinical data, was obtained from the North Denmark Region (journal no. 1-45-72-413-25).
